# Protection against HEMA-Induced Mitochondrial Injury *In Vitro* by Nrf2 Activation

**DOI:** 10.1155/2019/3501059

**Published:** 2019-04-07

**Authors:** Yang Jiao, Tao Niu, Huan Liu, Franklin R. Tay, Ji-hua Chen

**Affiliations:** ^1^Department of Stomatology, the 7th Medical Center of PLA General Hospital, Beijing 100700, China; ^2^The Affiliated Stomatological Hospital of Kunming Medical University, Kunming 650000, China; ^3^State Key Laboratory of Military Stomatology & National Clinical Research Center for Oral Diseases & Shaanxi Key Laboratory of Oral Diseases, Department of Prosthodontics, School of Stomatology, the Fourth Military Medical University, Xi'an 710032, China; ^4^Department of Endodontics, the Dental College of Georgia, Augusta University, Augusta, GA 30912, USA

## Abstract

Dental resin monomers such as 2-hydroxyethyl methacrylate (HEMA) disturb vital cell functions and induce mitochondrial intrinsic apoptosis via generation of oxidative stress. Nuclear factor erythroid 2-related factor 2 (Nrf2) regulates the gene expression of antioxidative enzymes and plays a crucial role in the maintenance of cellular redox equilibrium and mitochondrial homeostasis. The present study investigated the functional significance of Nrf2 in cellular response toward HEMA. It was found that HEMA stimulation promoted nuclear translocation of Nrf2 and increased Nrf2 and heme oxygenase-1 (HO-1) expression, which was further enhanced by Nrf2 activator *tert-*butylhydroquinone (tBHQ), but suppressed by Nrf2 inhibitor ML385. Pretreatment of primary human dental pulp cells (hDPCs) with tBHQ protected the cells from HEMA-induced oxidative injury (increased reactive oxygen species production and apoptosis) and mitochondrial impairment (morphological alterations, decreased ATP production, suppressed oxidative phosphorylation activity, depolarization of mitochondrial membrane potential, and disrupted electron transport chain). In contrast, pretreatment with ML385 increased cell sensitivity to these injurious processes. This protective effect on mitochondria could be related to peroxisome proliferator-activated receptor *γ* coactivator 1*α* (PGC1*α*)/nuclear respiratory factor 1 (NRF1) pathway. These results contribute to the understanding of the function of Nrf2 and the development of novel therapies to counteract the adverse effects of dental resin monomers.

## 1. Introduction

Significant concerns exist regarding the biological safety of resin-based dental materials despite their widespread use and long-term clinical success in contemporary dentistry. Incomplete polymerization and biodegradation of resin components result in the release of resin monomers such as 2-hydroxyethyl methacrylate (HEMA) into the oral environment, potentially causing local and/or systemic adverse effects [1, 2]. Because of their bioactivity, resin monomers are capable of diffusing rapidly through the dentinal tubules into dental pulp tissues [3, 4]. After the use of direct pulp capping procedures or restoration of deep cavities, pulpal responses including inflammation, dissociation of the odontoblastic cell layer, and necrotic cell death have been clinically observed [5, 6]. Along the dentin-pulp interface, bacterial and resin biodegradation by-products generate dynamic interactions and induce complex responses of the pulpal cells such as inhibition of lipopolysaccharide-stimulated NF-*κ*B activation [7]. The matrix-mineralizing capability or expression of genes essential for reparative dentin formation is disturbed in the odontoblasts [8, 9]. Resin monomers also induce apoptosis [10], autophagy [11], genotoxicity [12], and developmental toxicity [13]. The beneficial effect of antioxidants such as N-acetylcysteine [14–17] suggests that the mechanism behind these specific cell responses is the generation of oxidative stress [18].

Mitochondria are highly specialized and dynamic double-membrane organelles which regulate a host of cellular functions ranging from energy production and cellular metabolism to redox regulation and induction of intrinsic apoptosis [19]. Based on *in vitro* cell culture experiments using isolated mitochondria and cultured cells, resin monomers were found to disturb the integrity of mitochondrial structure and impair the bioenergetic functions of these organelles [15, 20]. Mitochondrial dysfunction exacerbates resin monomer-induced oxidative stress with activation of the mitochondrial apoptotic cascade [10, 15].

As the master regulator of the cellular redox homeostasis, the transcription factor nuclear factor erythroid 2-related factor 2 (Nrf2) promotes adaptation and survival under stressful conditions by regulating the expression of genes encoding proteins with diverse cytoprotective functions [21]. Under normal physiological conditions, Nrf2 is sequestered in the cytosol and maintained at a low level through Kelch-like ECH-associated protein 1- (Keap1-) mediated ubiquitination and proteasomal degradation. Upon cell exposure to oxidative stress, modifications of certain cysteine residues in Keap1 via disturbance of the ubiquitination and degradation of Nrf2 enable Nrf2 to accumulate and translocate to the nucleus. Within the nucleus, Nrf2 activates the expression of target genes through binding to an antioxidant-response element in the promoter region [22]. In addition to protecting against oxidative stress, carcinogenesis, and aging [23], Nrf2 is an important player in the maintenance of mitochondrial homeostasis and structural integrity [24, 25]. Enhanced expression of Nrf2 and differential expression of Nrf2-mediated antioxidative enzymes are examples of adaptive cellular responses to oxidative stress induced by resin monomers [15, 26].

In the present study, the authors hypothesized that Nrf2-mediated antioxidation and mitochondrial homeostasis may exert a protective function against resin monomer toxicity. To validate this hypothesis, the Nrf2 activator tBHQ and the Nrf2 inhibitor ML385 were used, respectively, to modulate Nrf2 expression. Primary human dental pulp cells (hDPCs) were examined for their cell viability and apoptosis, metabolic profiles, and mitochondrial alterations upon HEMA exposure. The null hypothesis tested was that Nrf2 expression has no influence on cell sensitivity toward HEMA and mitochondrial homeostasis.

## 2. Materials and Methods

### 2.1. Cell Culture and Treatment

The hDPCs were obtained from young healthy human subjects (18-25 years old) who had their noncarious third molars extracted. The procedure was reviewed and approved by the Ethics Committee of the 7th Medical Center of PLA General Hospital. After removal of dental pulp tissues from the tooth, the hDPCs were isolated and expanded as described in the authors' previous studies [14, 15, 27]. Briefly, the dental pulp tissues were minced and digested in a solution containing 3 mg/mL type I collagenase and 4 mg/mL dispase (Gibco, Grand Island, NY, USA) at 37°C for 2 h. Single-cell suspension was obtained by passing the cells through a 70 mm strainer (BD Falcon, Franklin Lakes, NJ, USA). The cells were then cultured in alpha-modified Eagle's medium (*α*-MEM; Gibco) supplemented with 10% fetal bovine serum (FBS; Gibco), 2 mM L-glutamine, 100 U/mL penicillin, and 100 g/mL streptomycin (Invitrogen, Carlsbad, CA, USA) at 37°C in a humidified atmosphere of 5% CO_2_. The culture medium was changed every 3 days, and passage 2–4 cells were used in all experiments. Based on the authors' recent investigation [15], oxidative stress in hDPCs was induced by treatment with 1 mM HEMA (Sigma-Aldrich, St. Louis, MO, USA) for the indicated times. To investigate the function of Nrf2 in HEMA-exposed cells, the hDPCs were pretreated with 25 *μ*M tert-butylhydroquinone (tBHQ; Sigma-Aldrich), a Nrf2 activator, for 18 h or 5 *μ*M ML385 (MedChemExpress, China), a Nrf2 inhibitor for 12 h, and subsequently cotreated with HEMA. The incubation concentrations and times of tBHQ or ML385 used in the present work were selected based on the authors' preliminary range-finding experiments and previous studies [26, 28].

### 2.2. Cell Viability

Cell viability was determined using a Cell Counting Kit 8 (CCK-8) Assay Kit (Beyotime, Jiangsu, China) according to the manufacturer's instructions. Briefly, hDPCs were seeded into 96-well plates at 5 × 10^3^ cells per well with a group of blank control wells (without cells) and a group of untreated control wells (cells exposed only to culture medium). Each incubation was performed in three separate cell culture wells. After resin monomer treatment, 10 *μ*L of kit reagent was added to 100 *μ*L of cell culture medium and incubated for 4 h at 37°C. Cell viability was obtained by monitoring the color change on a microplate reader (Bio-Rad, Hercules, CA, USA) with absorbance read at 450 nm.

### 2.3. Light Microscopy

Phase-contrast images were acquired using an Olympus IX70 microscope (Olympus, Tokyo, Japan). Three independent fields were acquired for each experimental condition. Representative samples from one field of view are shown.

### 2.4. Quantitative Real-Time Polymerase Chain Reaction

Total RNA was isolated using TRIzol reagent (Invitrogen) and reversely transcribed to cDNA using PrimeScript RT Reagent Kit (TaKaRa, Dalian, China). Quantitative real-time PCR (qRT-PCR) was performed using SYBR Premix Ex Taq II (Takara) in the Bio-Rad CFX96™ Real-Time System. The resulting amplification and melt curves were analyzed to ensure the identity of the specific PCR product. Threshold cycle values were used to calculate the fold change in the transcript levels by using the ^ΔΔ^CT method. Primers are listed in Table 1. The housekeeping gene *GAPDH* was used to normalize the expression level of related genes.

### 2.5. Western Blot Analysis

Cells were washed three times with phosphate-buffered saline (PBS). Proteins were extracted from the cells and quantified using a BCA Protein Assay Kit (Beyotime). Extracted proteins were loaded on 10% sodium dodecyl sulfate polyacrylamide gels, transferred to polyvinylidene fluoride membranes (Bio-Rad), and blocked with 5% nonfat milk powder. Membranes were incubated overnight with the following primary rabbit anti-human antibodies (Cell Signaling Technology, Beverly, MA, USA): anti-Nrf2 (1 : 2000), anti-HO-1 (1 : 2000), anti-NRF1 (1 : 2000), anti-PGC1*α* (1 : 2000), and anti-*β*-actin (1 : 5000). Membranes were incubated with goat anti-rabbit secondary antibodies. Protein signals were visualized using the ECL Western Blotting Detection System (GE Healthcare, Piscataway, NJ, USA). The gray values of the bands were analyzed using the ImageJ software (National Institutes of Health, Bethesda, MD, USA).

### 2.6. Immunofluorescence

Cells were fixed in 4% paraformaldehyde for 10 min, washed three times with PBS, permeated with 0.1% Triton X-100, and blocked with goat serum for 1 h. The cells were then incubated with primary antibodies (rabbit polyclonal anti-Nrf2, 1 : 100) overnight at 4°C. After washing with PBS, the cells were incubated with secondary antibodies (FITC-conjugated goat anti-rabbit, 1 : 200) for 1 h. The cells were then washed with PBS and further incubated with DAPI (1 : 1000, Dako, Glostrup, Denmark) for 15 min. Fluorescent images were obtained by an FV-1000/ES confocal microscope (Olympus).

### 2.7. Transmission Electron Microscopy

Cells were washed three times with PBS and fixed with 1% osmic acid at 4°C for 2 h. After dehydration in ethanol and embedding in epoxy resin, 70 nm thick sections were obtained using an ultramicrotome. The slices were stained with 3% uranyl acetate and citric acid and viewed with a transmission electron microscope (TEM; JEM-1230; JEOL, Tokyo, Japan).

### 2.8. Metabolic Activity

Oxidative phosphorylation (OXPHOS) activity was determined using an O_2_ Extracellular Sensor Kit (ENZO Life Sciences, Farmingdale, NY, USA) according to the manufacturer's instructions. After resin monomer treatment, the Extracellular O_2_ Sensor Probe was added to the hDPCs and measured using a fluorescence plate reader for 120 min, with fluorescence values of each time point normalized to the value obtained at 0 min.

The ratio of adenosine triphosphate (ATP) over adenosine diphosphate (ADP) was determined using an ADP/ATP Ratio Assay Kit (Abcam, Cambridge, MA, USA) according to the manufacturer's instructions. After resin monomer treatment, ATP and ADP levels were determined by measuring luminescence in the absence or presence of ADP-converting enzyme using a luminometer (Tecan, Switzerland) and the ATP/ADP ratio was subsequently calculated.

Intracellular ROS levels were determined using an ROS Assay Kit (Beyotime) according to the manufacturer's instructions. After resin monomer treatment, digested hDPCs were incubated with 2′7′-dichlorodihydrofluorescein diacetate and analyzed with flow cytometry (FACScan, Becton Dickinson, San Jose, CA, USA). A minimum of 10^4^ cells were analyzed per condition. Individual values of fluorescence intensity were normalized to the fluorescence detected in untreated control cultures.

Mitochondrial membrane potential (MMP) was determined using the fluorescent dye JC-1 (Beyotime) according to the manufacturer's instructions. After resin monomer treatment, digested hDPCs were incubated with the JC-1 staining solution (5 mg/mL) for 20 min at 37°C and then rinsed with JC-1 staining buffer. The fluorescence intensity of JC-1 aggregates was detected at the excitation/emission wavelength ratio of 525/590 nm, and the fluorescence intensity of the JC-1 monomers was measured at 490/530 nm by flow cytometry. A minimum of 10^4^ cells were analyzed per condition.

### 2.9. Determination of Apoptosis

Apoptosis was identified using an Annexin V-FITC Apoptosis Detection Kit (Beyotime) according to the manufacturer's instructions. Briefly, hDPCs were harvested and washed with PBS, resuspended in Annexin V binding buffer, and stained with Annexin V-FITC and propidium iodide (PI) for 15 min. Fluorescence was determined by flow cytometry (FACScan). A minimum of 10^4^ cells were analyzed per condition.

### 2.10. Statistical Analysis

Data are represented as means ± standard deviations of each independent experiment (*n* = 3). Statistical significance was evaluated by Student *t*-test or one-way analysis of variance (ANOVA) followed by Tukey posttest in the GraphPad Prism software (San Diego, CA, USA) at *α* = 0.05.

## 3. Results

### 3.1. Expressions of Nrf2 and HO-1 in HEMA-Exposed hDPCs

**Figure 1:**
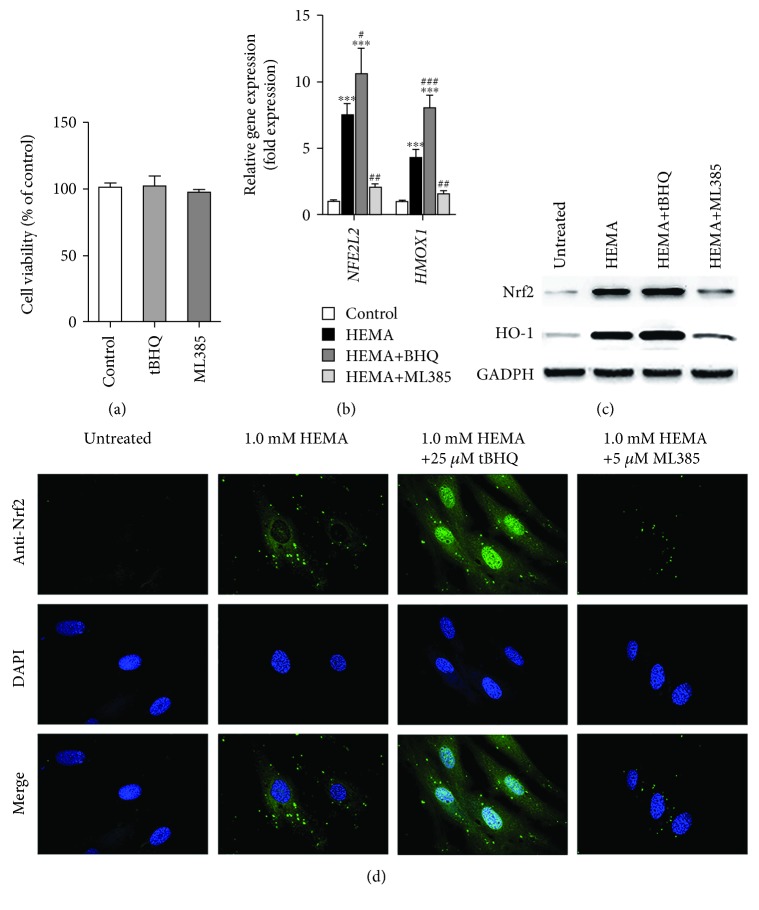
Expressions of Nrf2 and HO-1 in HEMA-exposed hDPCs. (a) Cytotoxicity of BHQ (25 *μ*M) and ML385 (10 *μ*M) after 24 h as evaluated by CCK-8 assay. No significant difference was observed among groups. (b) qRT-PCR analysis of mRNA expression levels of *NFE2L2* and *HMOX1* genes which encode Nrf2 and HO-1 proteins, respectively. (c) Western blot of Nrf2 and HO-1 protein expression levels. (d) Immunofluorescence analysis of Nrf2 expression and nuclear translocation. Green fluorescence represents Nrf2, and blue fluorescence represents nucleus of hDPCs. Data represent mean ± standard deviations (*n* = 3). ^∗^*P* < 0.05, ^∗∗^*P* < 0.01, and ^∗∗∗^*P* < 0.001 vs. untreated cells (control group); ^#^*P* < 0.05, ^##^*P* < 0.01, and ^###^*P* < 0.001 vs. HEMA-treated cells. Data were analyzed using one-way analysis of variance (ANOVA) and post hoc Tukey test.

The concentrations of the chemicals employed were first examined to identify if those concentrations were cytotoxic to hDPCs. When compared to the control group, tBHQ (25 *μ*M) and ML385 (10 *μ*M) have no cytotoxic effect on hDPCs (Figure 1(a)). The potentials for tBHQ and ML385 at their respective, selected noncytotoxic concentrations to modulate Nrf2 expression and related antioxidative enzymes in hDPCs exposed to 1 mM HEMA were subsequently evaluated. qRT-PCR analysis showed that the mRNA expression levels of *NFE2L2* and *HMOX1* genes, which encode Nrf2 and HO-1 proteins, in the HEMA experimental group were significantly higher than those in the control group (Figure 1(b)). Consistent with the qRT-PCR results, western blot analysis showed that expression levels of Nrf2 and HO-1 proteins were weak in untreated cells but drastically increased in cells exposed to 1 mM HEMA (Figure 1(c)).

The potential for the aforementioned factors to induce nuclear translocation of Nrf2 was then examined. Immunofluorescence analysis showed that Nrf2 protein was weakly detectable in the cell nuclei and cytosol of untreated cells. In contrast, Nrf2 expression increased extensively in the nuclei and cytosol of cells exposed to 1 mM HEMA; the observations suggested translocation of Nrf2 from the cytosol to the nucleus (Figure 1(d)). In addition, mRNA and protein expressions of both Nrf2 and HO-1 were upregulated by tBHQ, but downregulated in cells preincubated with ML385 (Figures 1(b)–1(d)). These results confirmed that tBHQ promoted nuclear translocation of Nrf2 and increased Nrf2 and HO-1 expression and that ML385 suppressed the Nrf2 pathway in HEMA-exposed cells.

### 3.2. *In Vitro* Sensitivity to HEMA Stimulation Was Nrf2-Dependent

**Figure 2:**
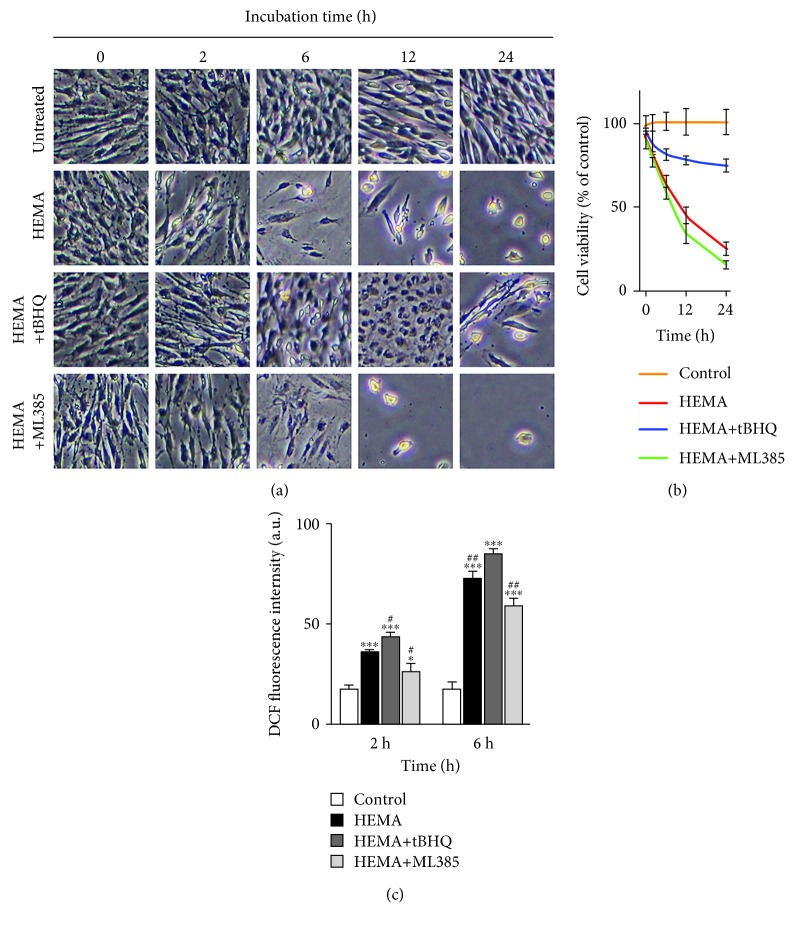
HEMA-induced cell death and ROS accumulation is related to Nrf2 expression. (a) Visualization of hDPC viability observed by phase-contrast microscopy. (b) Viability of hDPCs as examined by CCK-8 assay after 24 h. (c) Intracellular ROS level examined by flow cytometry after a 2 h or 6 h exposure period. Data represent mean ± standard deviations (*n* = 3). ^∗^*P* < 0.05, ^∗∗^*P* < 0.01, and ^∗∗∗^*P* < 0.001 vs. untreated cells (control group); ^#^*P* < 0.05, ^##^*P* < 0.01, and ^###^*P* < 0.001 vs. HEMA-treated cells. Data were analyzed using one-way analysis of variance (ANOVA) and post hoc Tukey test.

The notion that modulation of Nrf2 expression interfered with ROS level and related cell death in hDPCs exposed to HEMA was examined. Flow cytometry using 2′7′-dichlorodihydrofluorescein diacetate revealed that HEMA stimulation resulted in increased intracellular ROS levels beginning at 2 h (Figure 2(c)). This increase in ROS preceded cell detachment and overt death, which commenced at 6 h (Figure 2(a)). HEMA-induced ROS accumulation and cell death were suppressed by cotreatment with tBHQ, but were potentiated by ML385. After 24 h, cell viability was reduced to 25% by HEMA compared to the untreated cells and was further significantly decreased after suppressed Nrf2 expression by ML385. In contrast, upregulated Nrf2 expression by tBHQ significantly protected hDPCs from HEMA-induced cell death and cell viability increased to 75% (Figure 2(b)).

### 3.3. The Role of Nrf2 in HEMA-Induced Apoptosis

**Figure 3:**
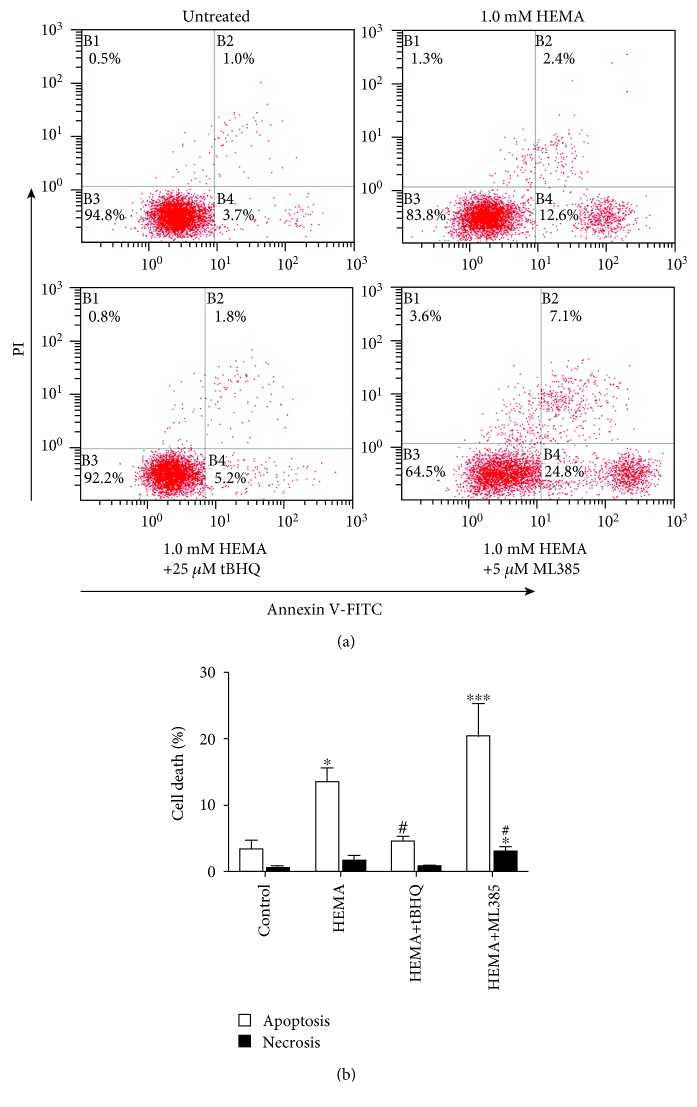
Induction of apoptosis and necrosis in HEMA-exposed hDPCs. After a 24 h exposure period, cells were stained with Annexin V-FITC (Annexin)/propidium iodide (PI) and analyzed by flow cytometry. (a) Percentages of viable cells (unstained, B3), and cells in apoptosis (Annexin, B4), late apoptosis (Annexin & PI, B2) and necrosis (PI, B1) of one typical experiment are denoted in the quadrants of each density blot. (b) Bar graphs represent the mean values of flow cytometry data. Data represent mean ± standard deviations (*n* = 3). ^∗^*P* < 0.05, ^∗∗^*P* < 0.01, and ^∗∗∗^*P* < 0.001 vs. untreated cells (control group); ^#^*P* < 0.05, ^##^*P* < 0.01, and ^###^*P* < 0.001 vs. HEMA-treated cells. Data were analyzed using one-way analysis of variance (ANOVA) and post hoc Tukey test.

The generation of oxidative stress has been shown to be causally related to resin monomer-induced apoptosis [15]. Accordingly, induction of apoptosis in hDPCs exposed to HEMA, with or without tBHQ or ML385 pretreatment, was further analyzed using flow cytometry. As shown in Figures 3(a) and 3(b), untreated cells exhibited minimal signs of apoptosis and more than 94.8% of the cells remained viable. Conversely, HEMA stimulation reduced the percentage of viable cells to 83.8%, and percentages of cells in the various phases of cell death, mostly in early apoptosis, were analogously increased to 12.6%. In the presence of tBHQ, there was an increase in the percentage of viable cells and a decrease in the percentage of cells undergoing late apoptosis or necrosis; the results were indicative of a well-defined protective effect of tBHQ on HEMA-induced apoptosis. In contrary, the presence of ML385 in HEMA-treated cells significantly reduced the percentage of viable cells to 64.5%, and the percentage of early apoptosis and necrosis of hDPCs increased to 24.8% and 3.6%, respectively. These results indicated that HEMA-induced apoptosis was increased when Nrf2 was inactivated but was partly inhibited when Nrf2 was activated.

### 3.4. Pretreatment with tBHQ Restored Mitochondrial Morphology and Function but ML385 Potentiated Mitochondrial Damage in HEMA-Exposed Cells

**Figure 4:**
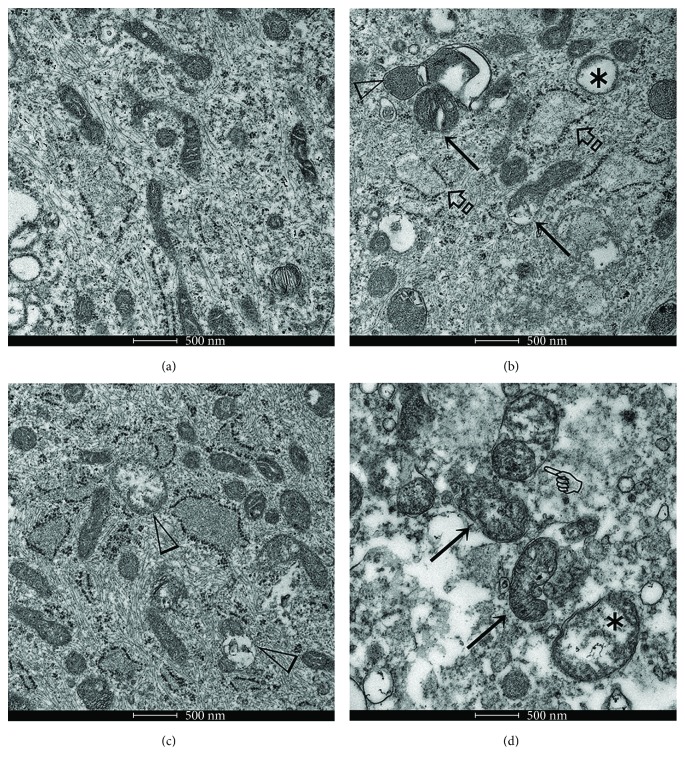
Transmission electron microscopy of mitochondrial morphological alterations before and after various treatments. (a) Well-preserved mitochondria in untreated hDPCs. (b) After the cells were exposed to HEMA, some mitochondria were swollen (arrows), with cristae derangements (open arrowhead) and vacuolization (asterisk). The rough endoplasmic reticulum also appeared swollen and disrupted (open arrowheads). (c) The presence of tBHQ partially restored mitochondrial morphology. However, some mitochondria were irreversibly damaged (open arrowheads). (d) Mitochondria exhibited extensive swelling and cristae derangements (arrows), disruption of membrane integrity (pointer), and internal vacuolization (asterisk) when the hDPCs were pretreated ML385 prior to exposure to HEMA. Bar = 500 nm.

The involvement of mitochondria in resin monomer toxicity has been well recognized [15, 20]. Hence, the function of Nrf2 in mitochondrial alterations and the related metabolic profiles of HEMA-exposed hDPCs were further investigated. Transmission electron microscopy of the control cells showed that the mitochondria had intact outer and inner membranes with well-defined cristae (Figure 4(a)). After HEMA treatment, some mitochondria were swollen with cristae derangements and internal vacuolization. The rough endoplasmic reticulum also appeared swollen and disrupted (Figure 4(b)). Conversely, tBHQ partially restored mitochondrial morphology, although some of the mitochondria were irreversibly damaged (Figure 4(c)). The use of ML385 further inflated the destructive effects of HEMA on mitochondria with deformed cristae, vacuolization, and loss in structural integrity (Figure 4(d)).

**Figure 5:**
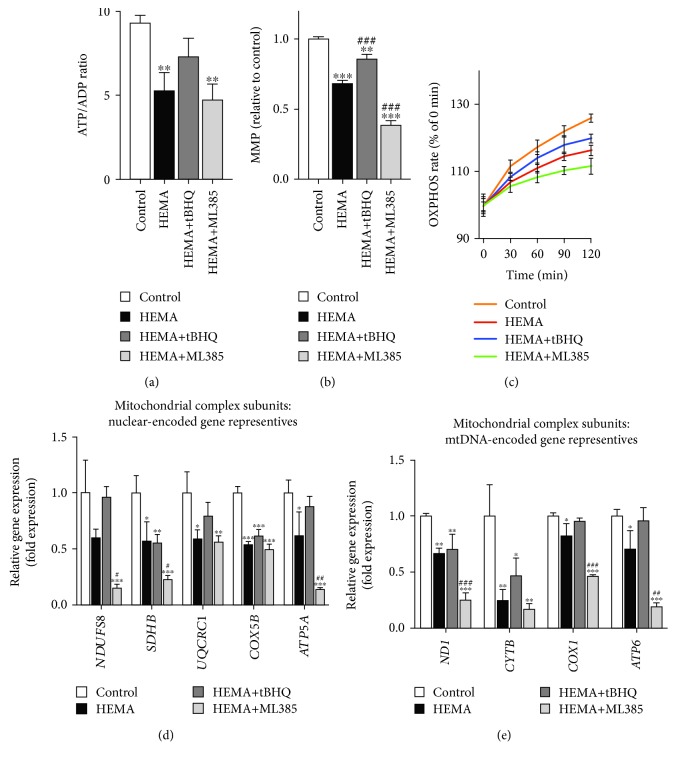
Nrf2 improves mitochondrial function in HEMA-exposed hDPCs. (a) Quantitative analysis of ATP production vs. ADP ratio. (b) JC-1 detection of mitochondrial membrane potential (MMP). (c) OXPHOS rate. (d, e) qRT-PCR analysis of mRNA expression levels of (d) nuclear-encoded and (e) mtDNA-encoded gene representatives for mitochondrial complex subunits of hDPCs. Data represent mean ± standard deviations (*n* = 3). ^∗^*P* < 0.05, ^∗∗^*P* < 0.01, and ^∗∗∗^*P* < 0.001 vs. untreated cells (control group); ^#^*P* < 0.05, ^##^*P* < 0.01, and ^###^*P* < 0.001 vs. HEMA-treated cells. Data were analyzed using one-way analysis of variance (ANOVA) and post hoc Tukey test.

As shown in Figure 5(a), the HEMA-exposed cells demonstrated significant reduction in ATP production when compared with untreated cells, which could be rescued by tBHQ pretreatment. The ATP/ADP ratio was reduced by ML385 in cells exposed to HEMA. tBHQ treatment also restored the suppressed OXPHOS activity of HEMA-exposed cells; in contrast, ML385 lowered OXPHOS activity even further (Figure 5(b)). As indicated by JC-1 staining (Figure 5(c)), exposure of cells to HEMA caused depolarization of mitochondrial membrane potential, which was further enhanced in the presence of ML385 but partially reversed by tBHQ. Based on such observations, metabolic changes in hDPCs after HEMA stimulation could be attributed to alterations in mitochondrial integrity.

The hypothesis of whether suppressed OXPHOS was the result of electron transport chain (ETC) disruption was further investigated. Considering that the ETC complex subunits are encoded by either the nuclear genome or mitochondrial DNA (mtDNA) [29, 30], representative genes encoding subunits of each of the ETC complexes were screened [31]. As shown in Figures 5(d) and 5(e), qRT-PCR showed that the mRNA expression levels of most of these genes were downregulated in cells exposed to HEMA. This trend was further enhanced in the presence of ML385 but partially reversed by tBHQ. Taken together, these results indicate that Nrf2 exerts a protective function to alleviate mitochondrial morphological alterations and dysfunction in cells exposed to HEMA.

### 3.5. Nrf2 Restored Impaired Mitochondria by Regulating the PGC1*α*/NRF1 Pathway

**Figure 6:**
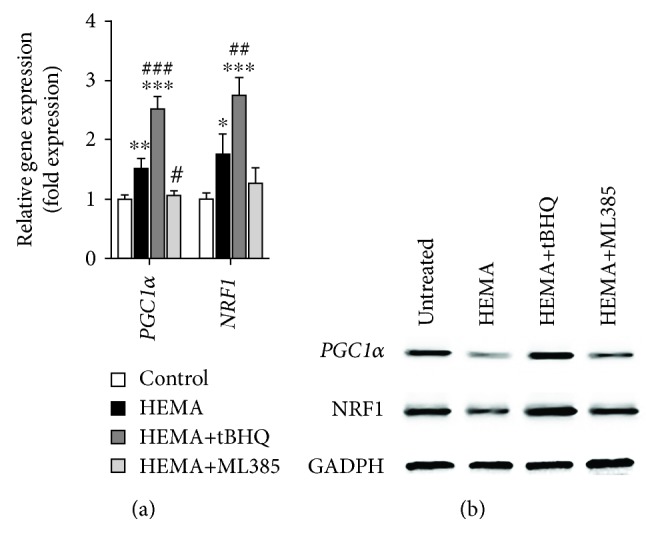
Nrf2 restored impaired mitochondria by regulating the PGC1*α*/NRF1 pathway in HEMA-exposed hDPCs. (a) qRT-PCR analysis of mRNA expression levels of *PGC1α* and *NRF1* genes. (b) Western blot of PGC1*α* and NRF1 protein expression levels. Data represent mean ± standard deviations (*n* = 3). ^∗^*P* < 0.05, ^∗∗^*P* < 0.01, and ^∗∗∗^*P* < 0.001 vs. untreated cells (control group); ^#^*P* < 0.05, ^##^*P* < 0.01, and ^###^*P* < 0.001 vs. HEMA-treated cells. Data were analyzed using one-way analysis of variance (ANOVA) and post hoc Tukey test.

To elucidate how Nrf2 restored mitochondria in HEMA-exposed cells, the expression levels of PGC1*α* and NRF1, which play central roles in a regulatory network that governs the transcriptional control of mitochondrial biogenesis and respiratory function [32], were further investigated. As shown in Figures 6(a) and 6(b), cells following HEMA stimulation showed decreased expression levels of PGC1*α* and NRF1 proteins but a significant increase of *PGC1α* and *NRF1* genes compared with untreated cells. Compared with the HEMA group, pretreatment with tBHQ significantly upregulated both mRNA and protein expression levels of PGC1*α* and NRF1, whereas ML385 further downregulated their expressions. These results indicate that Nrf2 potentially restores mitochondrial functions by regulating the PGC1*α*/NRF1 pathway in HEMA-exposed hDPCs, although detailed mechanism requires further investigation in depth in future studies.

## 4. Discussion

The functional significance of Nrf2 in the cellular response of hDPCs to the resin monomer HEMA was clearly demonstrated in the present study. By using chemicals that modulate Nrf2 expression, we found that pharmacological activation of Nrf2 had protective effects against HEMA-induced oxidative injury and mitochondrial impairment, whereas cells with inactivated Nrf2 were much more sensitive to these injurious processes. This protective effect on mitochondria could be related to the PGC1*α*/NRF1 pathway. These results indicate that modulation of the Nrf2-mediated cellular defense response is an effective strategy for manipulating the sensitivity of cells in the dental pulp to cytotoxic resin monomers.

Mitochondria are the major intracellular source of ROS but are also the most affected target of ROS [33]. In the present study, HEMA-induced mitochondrial morphological alterations were identified, which included elongated mitochondria and cristae derangements, as well as mitochondrial dysfunctions such as depolarization of MMP and decreased ATP production via OXPHOS. The majority of a cell's ATP is produced by the means of OXPHOS, which occurs in the mitochondrial cristae [34]. During this process, the energy released by electrons flowing through the ETC establishes an electrochemical proton gradient across the mitochondrial inner membrane, which is largely responsible for MMP. However, mitochondrial morphological alterations, especially deformed cristae structures, may result in the depolarization of MMP and the remaining proton gradient may not be sufficient to support a high rate of OXPHOS, as observed in the present study. Compromised mitochondrial function induced by resin monomers exacerbates ROS-related damage to cells of the dental pulp (e.g., odontoblasts and macrophages) via bioenergetic failure and intrinsic mitochondrial apoptosis [10, 15]. The ETC includes five protein complexes located in the mitochondrial inner membrane; complex (C) I to CIV mediate electron transport from the Krebs cycle coupled to the pumping of protons, and release of the generated electrochemical gradient through ATP synthase (CV) [35]. A recent study identified CI as a primary target of another common resin monomer triethylene glycol dimethacrylate (TEGDMA) [20]. However, HEMA caused ETC disruption by suppressing most of the ETC complexes, either by nuclear-encoded mitochondrial or mtDNA-encoded transcription [31]. This contradiction may be explained as follows: (i) in that study, isolated mitochondria were incubated with TEGDMA; (ii) in the course of resin monomer treatment, intracellular mitochondria and mitochondria obtained after isolation in situ may undergo structural and functional impairment to different extent; and (iii) although HEMA and TEGDMA shared similar mechanisms of toxicity, cells actively respond to different resin monomers by differential induction of cell death (e.g., HEMA but not TEGDMA induces autophagy in human gingival fibroblasts [11]).

Cells are equipped with an orchestrated antioxidative system to maintain cellular redox homeostasis; central to this cellular defensive machinery is the Nrf2 pathway [21]. In the present study, HEMA-stimulated hDPCs promoted nuclear translocation of Nrf2 and increased Nrf2 and HO-1 expressions. The high levels of Nrf2 expression could be attributed to disturbed Keap1-mediated degradation of Nrf2 by HEMA-induced oxidative stress [22] and/or direct reaction between the methacrylate group of HEMA with the cysteine residues within Keap1 [36]. Activation of Nrf2 counteracts the increase in ROS production by transcriptional upregulation of antioxidative enzymes such as superoxide dismutase (SOD), glutathione peroxidase (GPx), catalase (CAT), and HO-1 and also alleviates mitochondrial dysfunction. In a previous study, the authors showed that resin monomers induced the expression of SOD-1, GPx, and CAT, as a result of ROS overproduction and glutathione (GSH) depletion [15]. The function of these ROS-metabolizing proteins is assisted by HO-1 to provide auxiliary cytoprotective products (Fe, CO, and biliverdin) and glutathione reductase to recycle GSH [21, 22]. An RNA-Seq study in the same hDPC model identified the transcriptional activation of the coding *HMOX1* gene (encoding HO-1 protein) in response to clinically relevant low concentrations of TEGDMA [37].

Increases in the expression of Nrf2 and Nrf2-regulated antioxidative enzymes, including enzymes of directly metabolizing ROS or regulating GSH biosynthesis, as shown herein and in previous studies [15, 38], suggested the induction of cellular antioxidative defense. Nevertheless, mitochondrial damage and apoptosis were initiated through the formation of ROS exceeding the cellular antioxidative capacity; antioxidant substances such as N-acetylcysteine were shown to have protective effects [15]. Thus, we hypothesized that stimulation of the Nrf2 pathway might reduce oxidative stress and protect cells from toxicity caused by resin monomers [39]. To validate this notion, the Nrf2 activator tBHQ and inhibitor ML385 were used to modulate the expression of Nrf2. It was found that pharmacological activation of Nrf2 had protective effects against, whereas cells with inactivated Nrf2 were much more sensitive to HEMA-induced oxidative stress and apoptosis. These results further confirmed the indispensable function of Nrf2 in cellular antioxidative defense against cytotoxic resin monomers such as HEMA. Nrf2 is also critical for the maintenance of mitochondrial homeostasis and structural integrity. In the present work, pretreatment with tBHQ is capable of preventing mitochondrial morphological changes and dysfunctions induced by HEMA, while enhanced destructive effects in the mitochondria were observed in hDPCs with inactivated Nrf2. Several lines of evidence indicate that Nrf2 is involved in maintaining mitochondrial homeostasis in the stress response as part of its antioxidant function. For example, induction of Nrf2 by limb ischemic preconditioning confers resistance to opening of the mitochondrial permeability transition pore and mitochondrial swelling [40]. The hepatocytes of Nrf2-knockout mice, but not wild-type mice, showed swollen mitochondria with reduced cristae and disrupted membranes after a high-fat diet for 24 weeks [41]. Finally, to elucidate how Nrf2 restored mitochondria, the PGC1*α*/NRF1 pathway that plays a central role in regulating mitochondrial function, biogenesis, and respiration was investigated [42, 43]. Surprisingly, the authors found that expression levels of *PGC1α* and *NRF1* mRNA were upregulated compared with the control group after HEMA stimulation. However, the expression levels of PGC1*α* and NRF1 proteins were slightly reduced. This inconsistency between mRNA and protein expression levels might indicate that exposure to resin monomers caused mitochondrial biogenesis protein synthesis or assembly process dysfunction. The downregulation of NRF1 protein is also involved in dysfunctional synthesis of mitochondrial ETC complex subunits, resulting in reduced mitochondrial respiration function [44]. Therefore, decreased PGC1*α* and its downstream transcription factor NRF1 contribute to deficiency in mitochondrial oxidative capacity and energy production after HEMA stimulation. These results indicate that the resin monomer HEMA produces such an intense stress that it damages mitochondrial respiratory function, which is responsible for energy production by downregulating the mitochondrial biogenesis key regulator PGC1*α*/NRF1 pathway. The duration of the stress will in turn worsen the imbalance between energy production and energy demand to satisfy vital cell functions, which will lead to bioenergetic failure and apoptosis. In the present study, pretreatment with tBHQ significantly improves the activity of mitochondrial ETC complexes, and increases expression of key mitochondrial biogenesis factors PGC1*α* and NRF1. All of these findings indicate that Nrf2 protects hDPCs from HEMA-induced oxidative injury by improving mitochondrial respiratory function and mitochondrial biogenesis.

**Figure 7:**
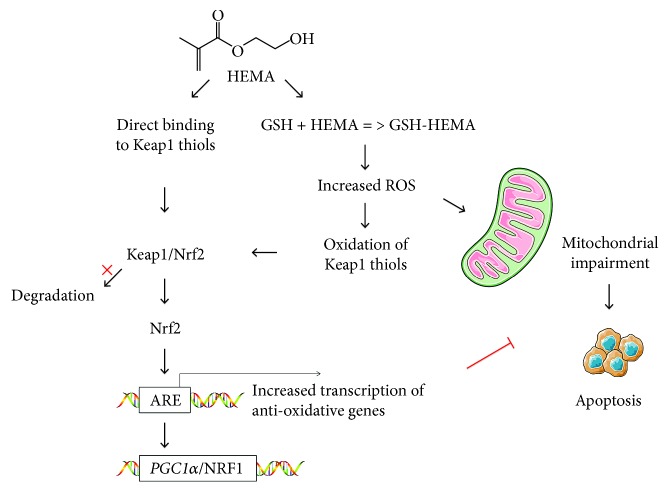
Proposed mechanisms for protection by Nrf2 against HEMA-induced cellular toxicity. Under physiological conditions, Nrf2 is sequestered in the cytosol and maintained at a low level through Keap1-mediated degradation. Resin monomers like HEMA disturb Keap1-mediated degradation of Nrf2 by increased oxidative stress and GSH depletion and/or by direct reaction with thiol groups of Keap1, to activate the Nrf2 pathway. Nrf2 neutralizes ROS and regenerates GSH, thus reducing ROS-mediated apoptosis and mitochondrial damage; Nrf2 restores impaired mitochondria by regulating the PGC1*α*/NRF1 pathway.

Based on the findings of the present study, the null hypothesis tested that Nrf2 expression has no influence on cell sensitivity toward HEMA and mitochondrial homeostasis has to be rejected. Under resin monomer-induced stress conditions, cells produce excessive ROS and deplete GSH, which elicits a multitude of responses. Reactive oxygen species directly damage macromolecules such as DNA, proteins, and lipids, resulting in mitochondrial dysregulation and further intracellular ROS production [18]. This ultimately induces mitochondrial damage and intrinsic apoptosis. The authors proposed mechanisms for protection by Nrf2 against resin monomer-induced cellular toxicity (Figure 7): Nrf2 neutralizes ROS and regenerates GSH, thus reducing ROS-mediated apoptosis and mitochondrial damage; Nrf2 restores impaired mitochondria by regulating the PGC1*α*/NRF1 pathway. Currently, the detailed mechanism by which Nrf2 maintains mitochondrial homeostasis under resin monomer-induced stress conditions is unclear and should be investigated in depth in future studies.

## 5. Conclusions

The present study investigated the therapeutic potential of activation of the Nrf2 pathway in HEMA-induced oxidative injury and mitochondrial impairment. Findings from the present work show that Nrf2 exerts a protective function through the alleviation of oxidative stress and maintenance of mitochondrial homeostasis. These results suggest that Nrf2 activation is a valuable therapeutic target for pharmacologic control of the adverse effects of dental resin monomers.

## Figures and Tables

**Table 1:** Primer sequences.

Category	Gene	Forward	Reverse
*Nrf2 pathway*			
	*NFE2L2*	5′-CTTGGCCTCAGTGATTCTGAAGTG-3′	5′-CCTGAGATGGTGACAAGGGTTGTA-3′
	*HMOX1*	5′-CAGGAGCTGCTGACCCATGA-3′	5′-AGCAACTGTCGCCACCAGAA-3′
*Nuclear encoded ETC subunits*			
NADH dehydrogenase (CI)	*NDUFS8*	5′-CGTCGAGGGCCCCAACTTT-3′	5′-TAGTCAGCCTGGATGTTGGC-3′
Succinate dehydrogenase (CII)	*SDHB*	5′-AAATGTGGCCCCATGGTATTG-3′	5′-AGAGCCACAGATGCCTTCTCTG-3′
Cytochrome B (CIII)	*UQCRC1*	5′-CAGTCCTCTCAGCCCACTTG-3′	5′-AAGCCAGATGCTCCAAAAAG-3′
Cytochrome C oxidase (CIV)	*COX5B*	5′-GTTTTGGCTGCACAAAGGGC-3′	5′-CTGGGGCACCAGCTTGTAAT-3′
ATP synthase (CV)	*ATP5A*	5′-AAGGAGATATAGTGAAGAGGACAG-3′	5′-ATAAGTCGTCATAGATGATCAAAGC-3′
*mtDNA-encoded ETC subunits*			
NADH dehydrogenase (CI)	*ND1*	5′-CTCAACTTAGTATTATACCC-3′	5′-GGAAATACTTGATGGCAGCT-3′
Cytochrome B (CII)	*CYTB*	5′TAGCAATAATCCCCATCCTCCATATAT-3′	5′-ACTTGTCCAATGATGGTAAAAGG-3′
Cytochrome C oxidase (CIV)	*COX1*	5′-ATTCGAGCAGAATTAGGTCA-3′	5′-CTCCGATTATTAGTGGGACA-3′
ATP synthase (CV)	*ATP6*	5′-TTTCCCCCTCTATTGATCCC-3′	5′-GTGGCCTTGGTATGTGCTTT-3′
*PGC1α/NRF1 pathway*			
	*PGC1α*	5′-GTAAATCTGCGGGATGATGG-3′	5′-AATTGCTTGCGTCCACAAA-3′
	*NRF1*	5′-CTACTCGTGTGGGACAGCAA-3′	5′-AATTCCGTCGATGGTGAGAG-3′
Control	*GAPDH*	5′-ATGACATCAAGAAGGTGGTG-3′	5′-CATACCAGGAATGAGCTTG-3′

## Data Availability

The data used to support the findings of this study are available from the corresponding author upon request.
